# Multiple Myeloma Presenting With Bronchopneumonia: A Case Report

**DOI:** 10.7759/cureus.56010

**Published:** 2024-03-12

**Authors:** Ashwin Karnan, Babaji Ghewade, Vivek D Alone, Anjana Ledwani

**Affiliations:** 1 Pulmonary Medicine, Datta Meghe Institute of Higher Education & Research, Wardha, IND; 2 Respiratory Medicine, Datta Meghe Institute of Higher Education & Research, Wardha, IND

**Keywords:** chemotherapy, protein electrophoresis, osteolytic lesion, m band, bone marrow, bence jones protein, hypercalcemia, myeloma

## Abstract

Multiple myeloma is a disease of the plasma cells of the bone marrow, resulting in the proliferation and release of the monoclonal protein, which further causes end-organ damage. We report an unusual presentation of multiple myeloma, thereby insisting on the need for the treating physician to be aware of the various presentations that can be encountered in regular practice. It is often difficult to diagnose, and the diagnosis is usually made at a late stage of the disease. Even though uncurable, with recent advances, a proper regimen, newer chemotherapeutic agents, and stem cell transplantation, the disease can be brought into remission.

## Introduction

Multiple myeloma is a cancer of the plasma cells of the bone marrow, resulting in the proliferation and release of the myeloma protein, which further causes end-organ damage. It accounts for 1.8% of all cancer cases per year in the US, with an incidence of 5.8/1 lakh population accounting for 20,000 new cases per year and an incidence of 0.7/1 lakh population accounting for 7,000 new cases per year in India [1]. The average age at diagnosis is 70 years and is slightly more common in males, with a male-to-female ratio of 1.4:1. Symptoms include bone pain, generalized tiredness, recurrent infections, easy bruising and bleeding, weight loss, and headaches. The prognosis depends on the stage of the disease. Diagnosis is usually made at the late stage of the disease. The five-year survival rate is around 59.8% [2], and survival rates have improved with the introduction of newer chemotherapeutic drugs and stem cell transplantation. Almost all cases of multiple myeloma go into repeated remission and relapse. Supportive treatment aimed at the correction of hypercalcemia, anemia, and pain also plays an important role.

## Case presentation

A 60-year-old female presented with complaints of breathing difficulty, dry cough, and chest pain for the preceding two days. The patient had been symptom-free until that point. The dry cough had an insidious onset and progressive nature, without positional variation. Breathlessness initially occurred on exertion, progressing to rest, and chest pain was diffuse and dull-aching with no radiation. The patient reported no similar past illnesses, no significant medical or family history, no history of addictions, and maintained a vegetarian diet.

Upon examination, the patient was conscious and oriented, with a height of 152 cm, a weight of 40 kg, and a BMI of 17.78 (underweight). The pulse rate was 120 beats/minute, the respiratory rate was 34 breaths/minute, and the blood pressure was 110/70 mm Hg (measured in the right upper arm in a sitting posture). Oxygen saturation was 75% at room air, improving to 96% with noninvasive ventilation (NIV) support (FiO2 100%, PEEP 5 cmH2O). In the respiratory system, bilateral crepitations were noted throughout the auscultation. The cardiovascular system exhibited audible S1 and S2 with no murmurs. There were no focal neurological deficits; abdominal pain and tenderness were absent, and lumbar region tenderness was noted.

**Figure 1:**
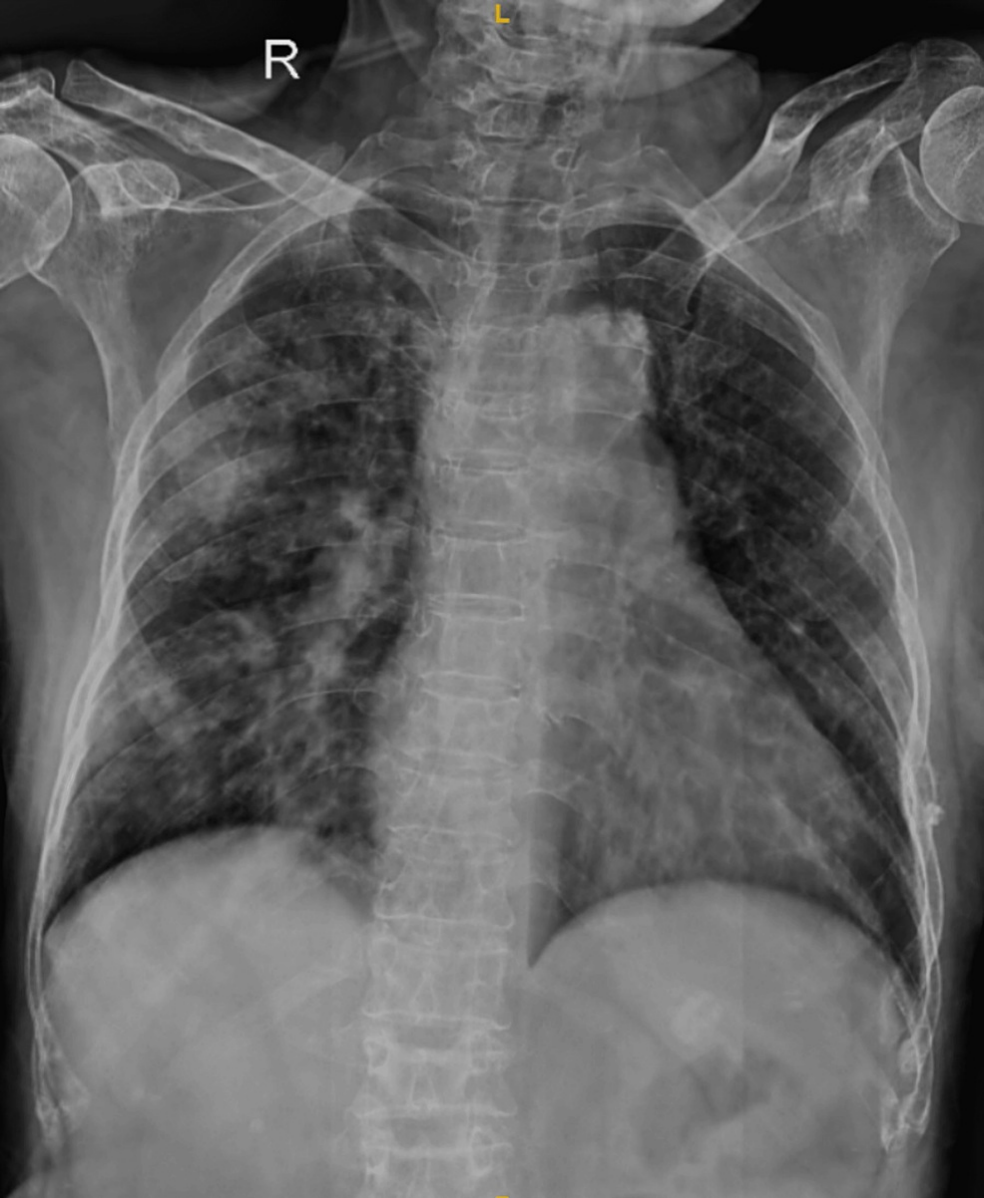
Chest X-ray showing bilateral bronchopneumonia

Arterial blood gas suggested type I respiratory failure. The 12-lead ECG was within normal limits. The chest X-ray showed bilateral inhomogeneous shadows (Figure 1).

**Figure 2:**
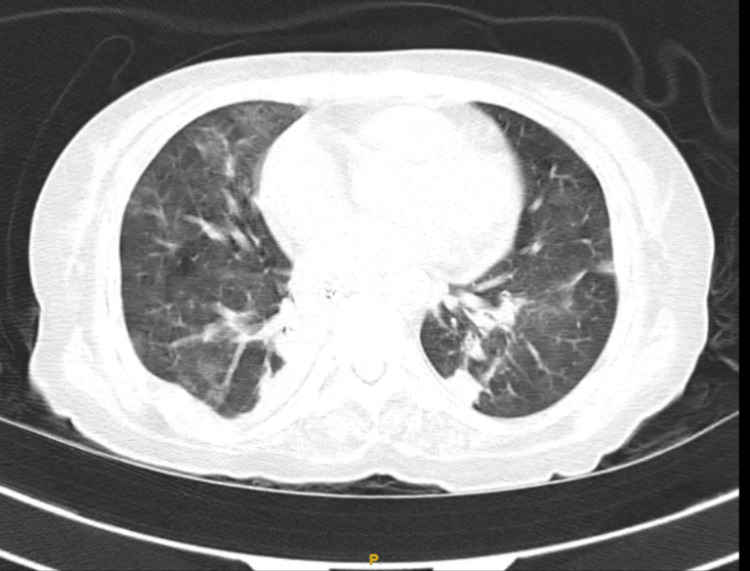
HRCT thorax showing bilateral patchy consolidation with cardiomegaly and bilateral diffuse ground glass opacity suggestive of pulmonary edema HRCT, high-resolution computed tomography

High-resolution computed tomography of the thorax showed bilateral patchy consolidation with cardiomegaly (Figure 2).

**Figure 3:**
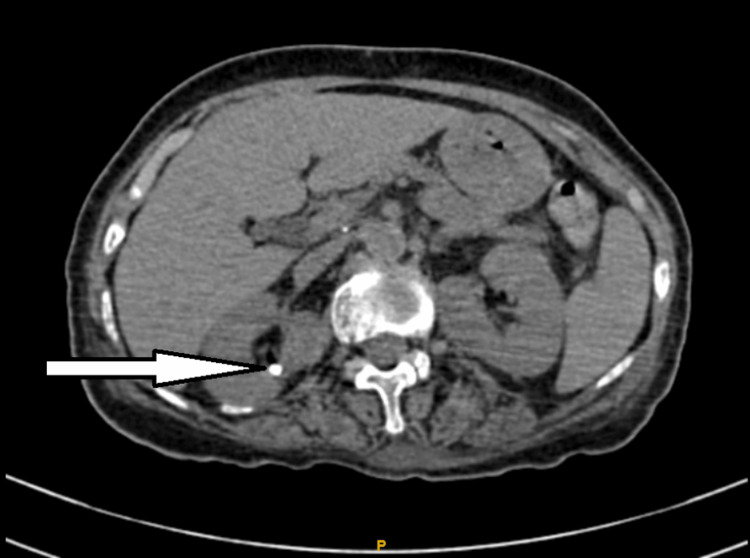
CT scan showing degenerative changes in the spine and hyperdense calculi in the right kidney CT, computed tomography

A computed tomography of the abdomen and pelvis was performed, which showed hyperdense calculi in the right kidney (Figure 3).

The complete blood count of the patient showed normocytic normochromic anemia (Table 1).

**Table 1:** Complete blood count showing normocytic normochromic anemia Hb, hemoglobin; HCT, hematocrit; MCH, mean corpuscular hemoglobin; MCHC, mean corpuscular hemoglobin concentration; MCV, mean corpuscular volume; RBC, red blood cell; WBC, white blood cell

Investigations	Values	Reference range
Hb	9.2 g/dL	13-17 g/dL
MCHC	33.5 g/dL	30-35 g/dL
MCV	85.6 fl	80-100 fl
MCH	28.7 Pico-gm	27-32 Pico-gm
Total RBC count	3.2 million/mm^3^	4.5-6 million/mm^3^
Total WBC count	13,400/mm^3^	4,000-11,000/mm^3^
Total platelet count	1.12 lacs/mm^3^	1.50-4.10 lacs/mm^3^
HCT	27.40%	Male: 41-50%; female: 36-44%

The liver function test of the patient showed elevated globulin levels with low serum albumin (Table 2).

**Table 2:** Liver function test showing raised globulin and low albumin ALP, alkaline phosphatase; ALT, alanine transaminase; AST, aspartate transferase; SGOT, serum glutamic-oxaloacetic transaminase; SGPT, serum glutamic pyruvic transaminase

Investigations	Values	Reference range
ALP	116 U/L	38-126 U/L
ALT (SGPT)	22 U/L	Male: <50 U/L; female: <35 U/L
AST (SGOT)	54 U/L	15-46 U/L
Total protein	11.0 g/dL	6.3-8.2 g/dL
Albumin	3.0 g/dL	3.5-5.0 g/dL
Globulin	8.0 g/dL	2.3-3.5 g/dL

The renal function test showed raised urea and creatinine with raised serum calcium (Table 3).

**Table 3:** Deranged renal function test with elevated serum calcium

Investigations	Values	Reference range
Urea	90 mg/dL	Male: 9-20 mg/dL; female: 7-17 mg/dL
Creatinine	2.0 mg/dL	Male: 0.66-1.25 mg/dL; female: 0.52-1.04 mg/dL
Calcium	14.0 mg/dL	8.1-10 mg/dL

**Figure 4:**
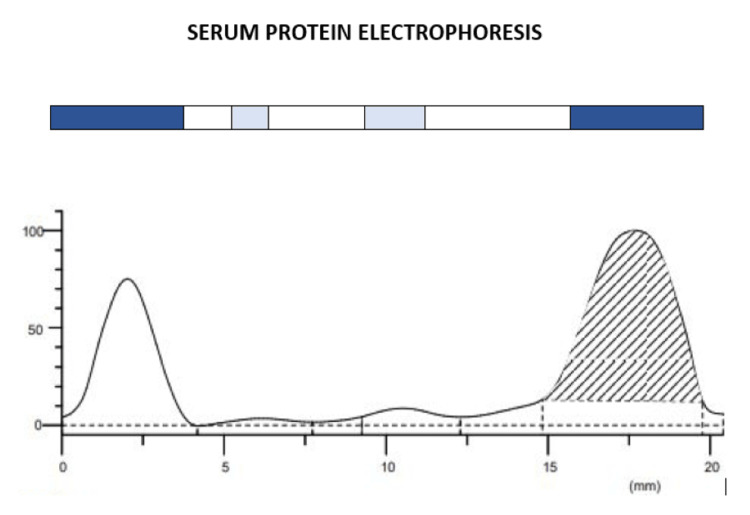
Serum protein electrophoresis showing a spike in the gamma globulin region with an M band suggestive of multiple myeloma

Serum protein electrophoresis showed a spike in the gamma M band, and a serum free light chain assay showed raised levels of free lambda light chain (Figure 4, Table 4).

**Table 4:** Serum protein electrophoresis report showing an elevated gamma globulin level

Index	Band	Percentage	Concentration (g/dL)	Range (g/dL)
1	Albumin	28.34%	3.32 L	3.50-5.00
2	Alpha 1	1.45%	0.17	0.10-0.40
3	Alpha 2	0.73%	0.08 L	0.5-1.10
4	Beta	3.86%	0.45 L	0.60-1.30
5	Gamma	65.62%	7.68 H	0.60-1.60
	Total 11.70			
5M	Gamma	49.06%	5.74 H	

**Figure 5:**
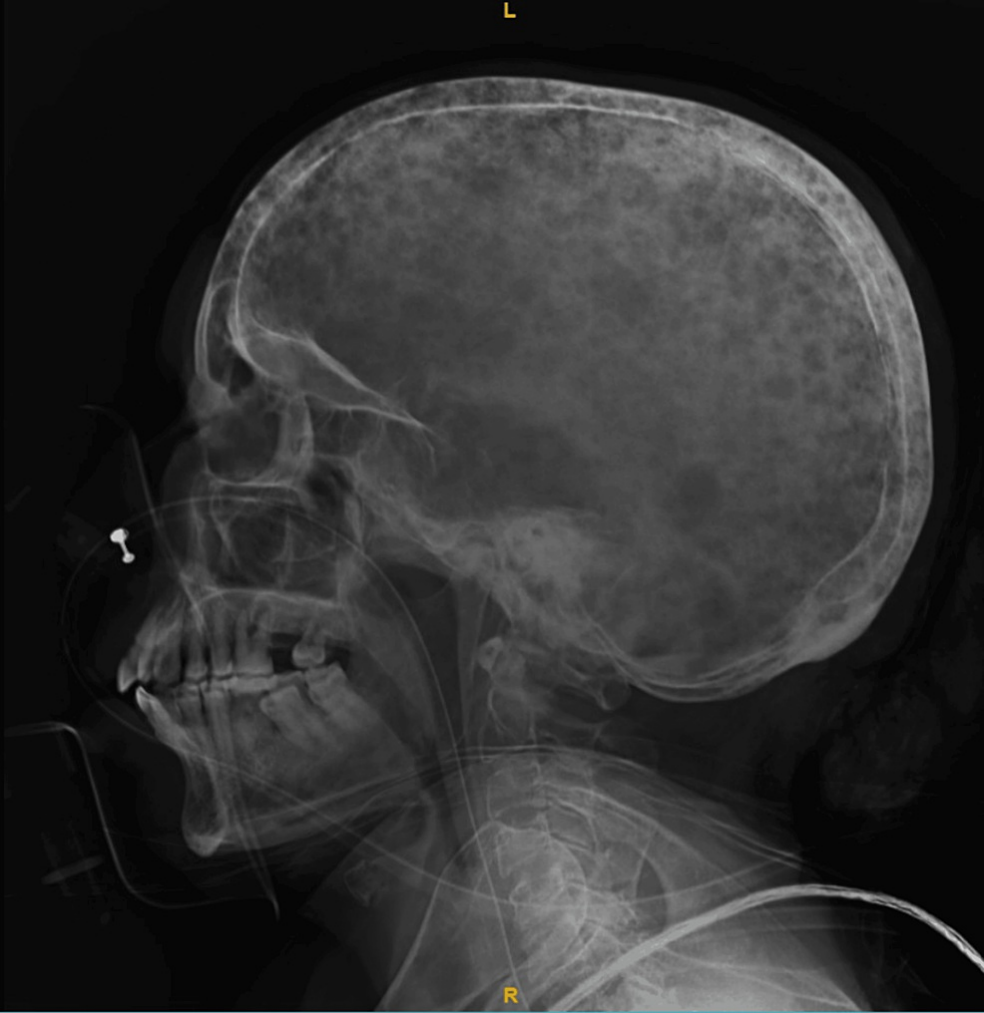
Lateral view of the skull showing multiple punched-out lytic lesions referred to as raindrop skull

An X-ray of the skull showed lytic lesions (Figure 5).

The bone marrow aspirate showed normocytic normochromic anemia with Rouleaux formation and numerous immature plasma cells with irregular nuclei.

The patient was treated with intravenous antibiotics, intravenous fluids, non-NIV support, and chest physiotherapy. She was then shifted to the oncology department for chemotherapy.

## Discussion

Multiple myeloma is characterized by the proliferation of cancerous plasma cells that grow out of control, produce an abnormal antibody called M protein, and cause end-organ damage. The plasma cells activate osteoclastic activity, causing bone destruction (pain, fracture, and immobility). It accounts for 1.8% of cancer cases in the US and 1.1% of all cancer deaths. Risk factors include advancing age, male sex, which is twice as common among African Americans, and people with a family history. Genetic alteration in the form of chromosomal deletion (del(17p)) and translocation (t(14;16), t(11;14), t(4;14)) is known to initiate disease and cause progression. These genetic alterations alter signaling pathways, which lead to plasma cell growth and proliferation. These plasma cells interact with the microenvironment of the bone marrow and induce osteoclastic activity [3]. Clinical manifestations include tiredness, excessive thirst, bone pain, easy bruising, recurrent infections, bleeding manifestations, and weight loss [4]. Diagnostic criteria include (1) clonal bone marrow plasma cells >10% or biopsy-proven extramedullary plasmacytoma, along with (2) evidence of end-organ damage due to plasma cell proliferative disorder such as hypercalcemia >11 mg/dL, renal failure creatinine >2 mg/dL, anemia (hemoglobin <10 g/dL), or one or more osteolytic lesions on skeletal radiography or clonal bone marrow >60% or uninvolved serum free light chain ratio >100 [5].

The five-year survival rate of multiple myeloma is around 57%, and the prognosis has increased due to therapeutic advances [6]. Multiple myeloma should be classified into high- or standard-risk groups, and proper treatment regimens should be initiated in such a way that future treatment is unaffected. If the patient is fit for transplant, the patient is treated with three to four cycles of monoclonal antibody/immunomodulator with a low-dose steroid; if a good response is noted, the patient can avoid maintenance therapy [7]. A trial involving bortezomib, dexamethasone, and lenalidomide showed a good response rate and survival rate, making it the initial therapy for newly diagnosed cases [1]. Along with chemotherapy, supportive treatment to correct hypercalcemia, anemia, and bone pain is important, which includes regular treatment with bisphosphonates [8]. Despite treatment options, multiple myeloma is known to have multiple remissions and relapses. While treating a case of relapse, time of relapse, aggressiveness, performance score, and response to earlier treatment are taken into account [9].

## Conclusions

Multiple myeloma is a rare, aggressive neoplastic disease that is on the rise in developing countries. Although incidence has risen, survival rates have increased due to newer drugs and stem cell transplant options. Bronchopneumonia in an immunocompromised patient is a complex infection with high mortality and morbidity. It may be further complicated by sepsis, multidrug resistance, pneumothorax, drug side effects, and respiratory distress. Since multiple myeloma is a complex disease, patient education and counseling are very important. As there is multisystem involvement, a multidisciplinary approach and a lookout for unusual presentations should always be kept in mind. In addition to chemotherapy, pain management, physiotherapy, diet, and nutrition should be given equal importance.
